# Technological Processes for Increasing the Cavitation Erosion Resistance of Nimonic 80A Superalloys

**DOI:** 10.3390/ma16083206

**Published:** 2023-04-18

**Authors:** Cosmin Belin, Ion Mitelea, Ilare Bordeașu, Corneliu Marius Crăciunescu, Ion-Dragoș Uțu

**Affiliations:** 1 Department of Materials and Fabrication Engineering, Politehnica University Timisoara, Bulevardul Mihai Viteazul nr.1, 300222 Timisoara, Romania; cosmin.belin@student.upt.ro (C.B.); ion.mitelea@upt.ro (I.M.); corneliu.craciunescu@upt.ro (C.M.C.); 2 Department of Mechanical Machines, Equipment and Transports, Politehnica University Timisoara, Bulevardul Mihai Viteazul nr.1, 300222 Timisoara, Romania; ilare.bordeasu@upt.ro

**Keywords:** Nimonic 80A, cavitation erosion, alternative technologies

## Abstract

Nickel-based superalloys are frequently used to manufacture the components that operate under cavitation erosion conditions, such as aircraft gas turbine construction, nuclear power systems, steam turbine power plants, and chemical and petrochemical industries. Their poor performance in terms of cavitation erosion leads to a significant reduction in service life. This paper compares four technological treatment methods to improve cavitation erosion resistance. The cavitation erosion experiments were carried out on a vibrating device with piezoceramic crystals in accordance with the prescriptions of the ASTM G32—2016 standard. The maximum depth of surface damage, the erosion rate, and the morphologies of the eroded surfaces during the cavitation erosion tests were characterized. The results indicate that the thermochemical plasma nitriding treatment can reduce mass losses and the erosion rate. The cavitation erosion resistance of the nitrided samples is approximately 2 times higher than that of the remelted TIG surfaces, approximately 2.4 times higher than that of the artificially aged hardened substrate, and 10.6 times higher than that of the solution heat-treated substrate. The improvement in cavitation erosion resistance for Nimonic 80A superalloy is attributed to the finishing of the surface microstructure, graining, and the presence of residual compressive stresses, factors that prevent crack initiation and propagation, thus blocking material removal during cavitation stresses.

## 1. Introduction

Nickel-based superalloys are engineering materials whose mechanical properties can be improved by alloying, by the kinetics of solidification processes, and by thermal and thermochemical treatments [1,2]. They are intended for the manufacturing of components of aircraft gas turbines, steam turbine power plants, components of the nuclear energy production industry, the chemical and petrochemical industry, and other hydromechanical equipment [2].

Such components are frequently exposed during operation to cavitation erosion phenomena. Cavitation is the process of the formation, growth, and implosion of bubbles containing vapor, gas, or vapor–gas mixtures due to cyclic pressure changes in the flowing liquid [2,3,4,5,6,7,8].

The cavitation phenomenon involves the interaction of hydrostatic, mechanical, metallurgical, and chemical processes. This notion includes the formation and subsequent rupture of voids in liquids, which are caused by the local drop in the hydrostatic pressure below a critical value, which is the saturation vapor pressure. The momentary decreases of the local pressure can be caused, on the one hand, by the spontaneous increase in the flow speed, and on the other hand, by an expansion of the shock waves during the low-pressure phase.

The voids contain the vapor phase of the liquid with or without parts of the gases that have been dissolved in it [1].

The research carried out so far has shown that the level of cavitation damage in metallic alloys depends, on the one hand, on the cavitation intensity, which is specific to the hydro-dynamics of the cavitation current, and, on the other hand, on the nature and physical condition of the material that produces a certain influence [1,2,3,4]:

- Chemical composition, respectively the nature and amount of alloying elements;
- Semi-finished product development technology (cast, laminated, etc.);
- Surface hardening treatment (thermal, thermomechanical, thermo-chemical, etc.);
- The values of the mechanical properties (Rm, Rp0.2, HV, KCU);
- The morphology of the microstructural constituents.

In order to improve the cavitation erosion resistance, research efforts are oriented towards the application of volume or surface thermal treatments [2,5,7,8], as well as some techniques of physical surface modification (welding deposition [9,10], cathodic arc plasma deposition [6], laser shock peening [11], and laser or TIG surface remelting [12,13], etc.). In many cases, the coatings developed by these techniques show a series of drawbacks, among which the presence of porosities, low adhesion, dilution, and inhomogeneous microstructure are mentioned. Due to these defects, the performances of the deposited layers in terms of cavitation erosion are limited. The technical literature contains numerous data on the cavitation erosion behavior of stainless steels, alloyed cast irons, cobalt-based alloys, and shape memory alloys, but few data on the cavitation erosion of nickel-based alloys [2,9,14,15]. Mesa D.H. et al. [8] investigated the effect of a composite surface treatment, which consisted of a high-temperature gas nitriding (HTGN) followed by a low-temperature plasma nitriding (LTPN), applied to a Duplex stainless steel UNS S31803, on the strength of cavitation erosion. The composed treatment caused an almost 9-fold increase in the incubation time, a phenomenon due to the mechanical resistance and higher hardness of the surface layer. Chang J.T. et al. [3] demonstrated that the deposition of a Ni-Al film on AISI 1045 carbon steel using the cathodic arc plasma (CAP) ion plating process is beneficial for cavitation erosion resistance. Chollet S. et al. [5] investigated the microstructure of Ni-based superalloys, either monocrystalline or polycrystalline, subjected to plasma nitriding, highlighting the variation in the thickness of the nitrogen-enriched layer depending on the chemical composition of the alloy and the duration of the thermochemical treatment.

The present contribution investigates the cavitation erosion response of a nickel base superalloy subjected to different hot manufacturing processes (plasma nitriding, TIG surface remelting, and solution treatment with or without artificial aging).

## 2. Researched Material

The research material is Nimonic 80A alloy (EN NiCr20TiAl; UNS No 7080; W. Nr. 24952& 24631), with the chemical composition listed in Table 1.

The main alloying element is chromium, which improves the resistance to hot oxidation. Additions of aluminum and titanium further favor the strengthening of the alloy through aging. The semi-finished products made of this alloy are delivered either in a solution heat-treated state or in an artificial aged state. The best mechanical properties of the Nimonic 80A alloy are obtained by applying the solution treatment followed by artificial aging.

**Figure 1:**
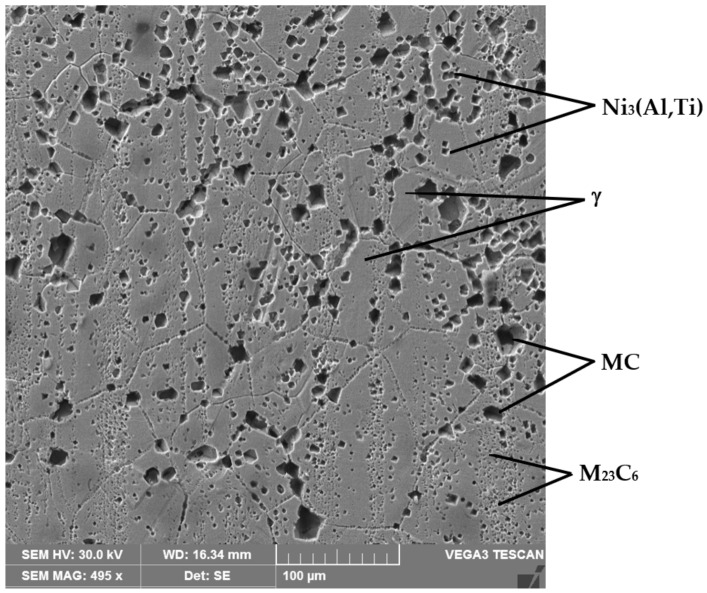
The microstructure of the Nimonic 80A alloy solution heat-treated followed by artificial aging.

The microstructure given in Figure 1 is made up of a solid solution matrix γ with a Ni base, of primary carbides, MC, with dimensions up to 20–30 µm, arranged intragranularly, that are rich in Cr of the M_23_C_6_ type with dimensions of a few 100 nm, arranged intergranularly and from precipitations of intermetallic phases γ′ of the Ni_3_(Al, Ti) type, whose size is ≈40–60 nm), inside the matrix grains. These observations fully agree with the results of other research works [5,16].

## 3. Experimental Stand and Work Procedure

**Figure 2:**
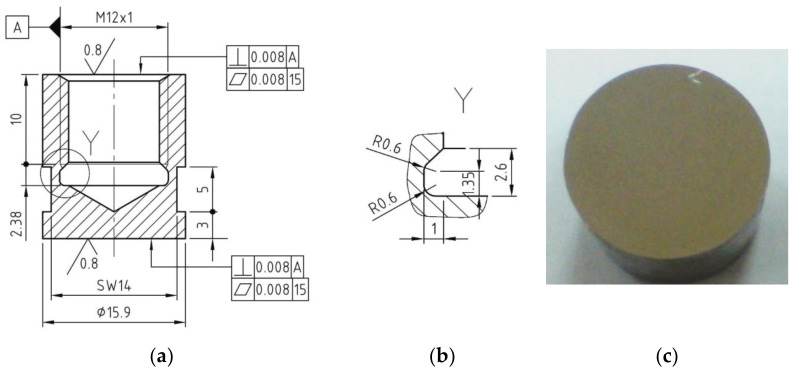
Geometry of cavitation samples: (**a**) shape and sizes; (**b**) manufacturing detail; (**c**) surface before cavitation.

**Figure 3:**
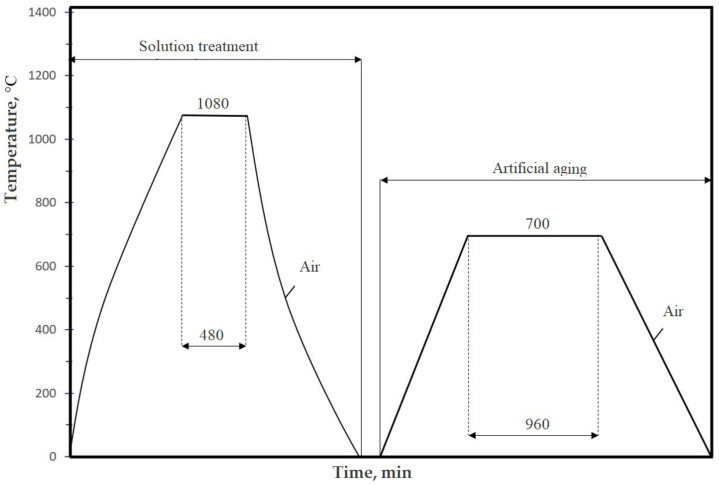
Cyclogram of the solution heat treatment followed by artificial aging.

From Nimonic 80A superalloy delivered in the form of cylindrical bars with a diameter of 20 mm, cavitation samples were made (Figure 2), which were subjected to the following technological processing:

- Solution treatment, 1080 °C/8 h/air;
- Solution treatment followed by artificial ageing, 700 °C/16 h/air (the cyclogram shown in Figure 3);
- Solution treatment followed by surface local remelting using the TIG technique;
- Solution treatment followed by plasma nitriding at 530 °C/14 h.

**Figure 4:**
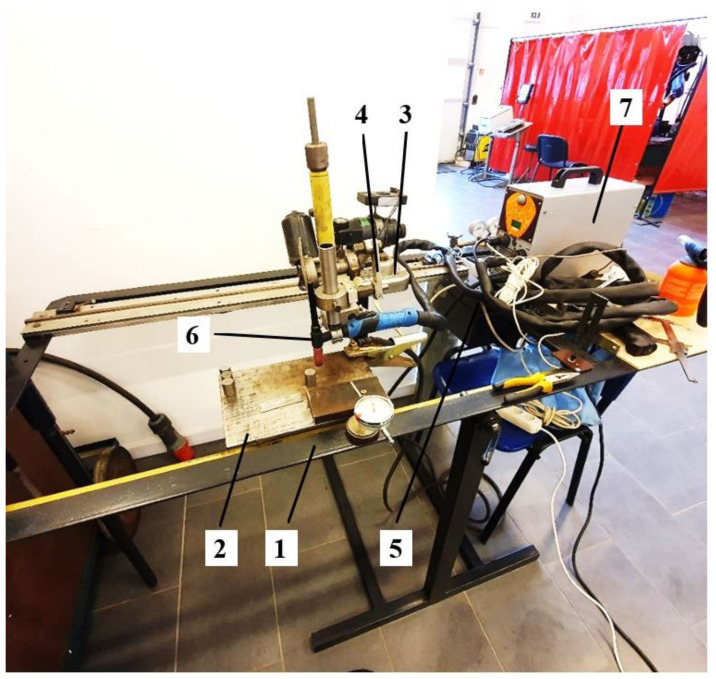
Overview of the TIG remelting stand: 1 metal frame; 2 piece positioning plate; 3 displacement device; 4 oscillation device; 5 power supplies of displacement–oscillation devices; 6 TIG torch; 7 welding source ESAB ET 300 iP.

The components of the experimental stand used for the local surface TIG remelting are shown in Figure 4. The mobility, which provided to the welding source in order to allow the correct positioning of the samples on the work table was achieved with the help of a movement-pendulum device built for this purpose.

**Figure 5:**
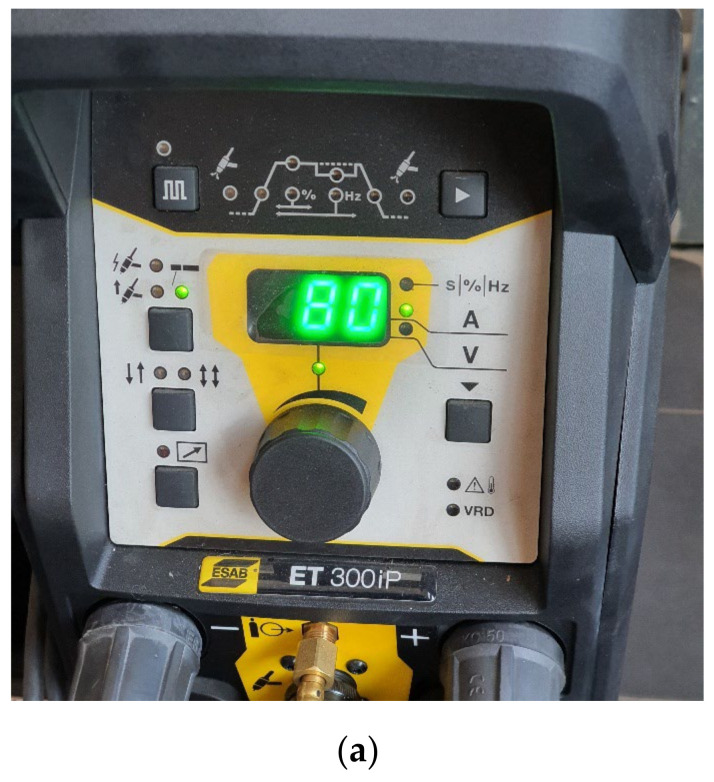

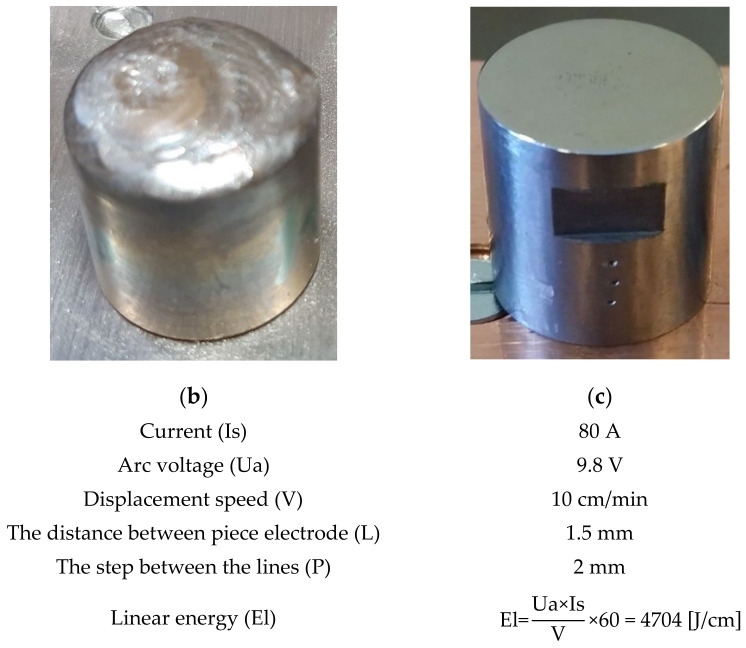
The control panel of the welding source (**a**), the aspect of the cavitation sample with the TIG remelted surface (**b**), with the surface prepared for testing (**c**), and the TIG remelting parameters.

Previous research [17] established that the optimal values of the melting current of the heat input that ensure the highest performance of cavitation erosion resistance are as follows: Is = 80 A, El = 4704 J/cm. The parameters of the local TIG surface remelting process together with the macroscopic image of the sample in the two stages (remelted surface, respectively later ground and polished) are shown in Figure 5.

**Figure 6:**
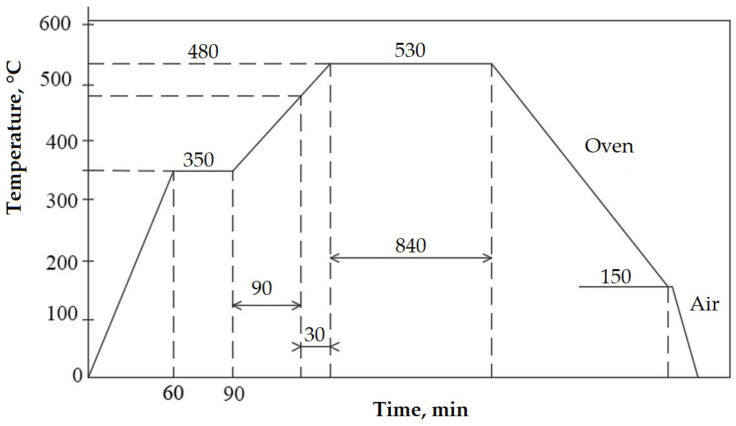
Cyclogram of thermochemical plasma nitriding treatment [18].

The thermochemical plasma nitriding treatment was carried out in a furnace equipped with a PROTHERM 500 (Mississauga, Ontario L5L 5Y7 Canada) controller. The cystogram of this treatment is shown in Figure 6 [17]. The samples were introduced into the retort of the installation and preheated to 350 °C for 30 min. This was followed by the introduction of ammonia and the continuation of the heating—holding phases up to the operating temperature of 530 °C. From 480 °C, the dissociation of ammonia practically started with the release of active nitrogen atoms. The cooling of the samples was carried out in the treatment oven up to a temperature of 150 °C, at which time they were taken out into the air.

The acoustic cavitation of these treated samples was performed using an ultrasonic emitter according to the G32-2016 standard of the American Society for Testing and Materials (ASTM) [19]. The oscillating pressure waves generated by the ultrasonic vibrations cause numerous bubbles to rise and collapse. The bubbles cluster in the cavity, and the sample can oscillate with a frequency lower than the excitation frequency, i.e., subharmonic oscillation. Previous studies on this material [17,18] have shown that ultrasonic cavitation erosion results in plastic deformation, initiation and crack propagation, partial material removal, and ultimately part failure.

**Figure 7:**
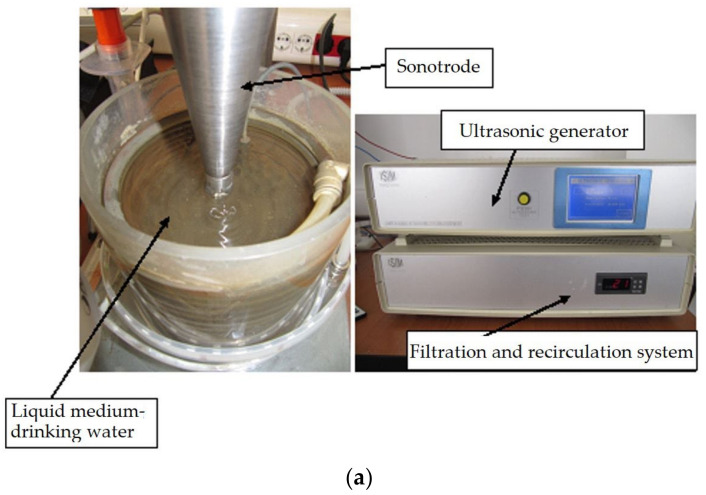

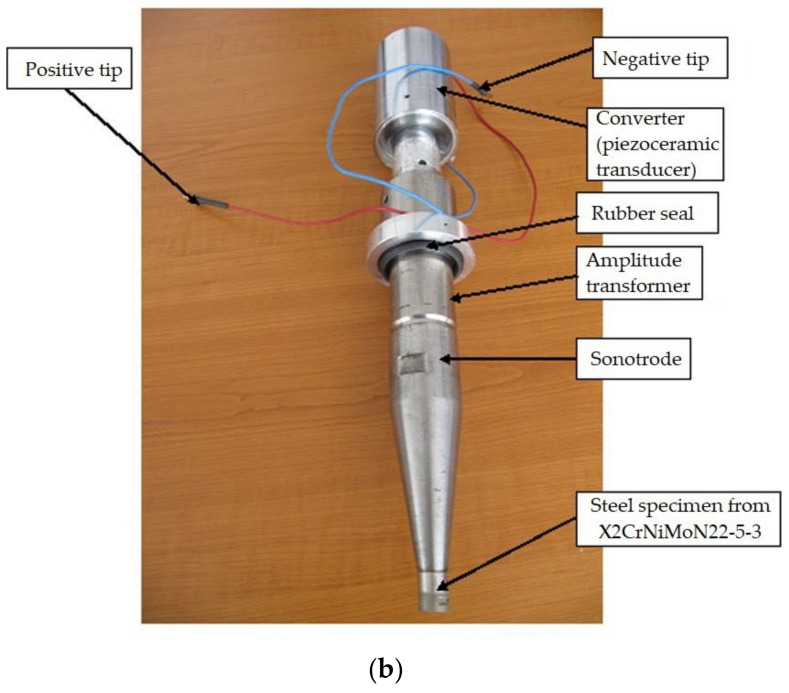
Image of the sample in the cavitation process (**a**) and of the mechanical resonator assembly (**b**).

Figure 7 shows the detailed image of a vibrating device with piezoceramic crystals used in the experimental program. The functional parameters of the device are as follows:

- Electronic ultrasonic generator power, 500 W;
- Vibration frequency, 20,000 ± 2% Hz;
- Vibration amplitude, 50 μm;
- Sample diameter: 15.8 ± 0.05 mm;
- Power supply: 220 V/50 Hz;
- Working media: drinking water from the industrial network, with a temperature of 22 ± 1 °C.

Before cavitation testing, the exposed surface of each sample was polished on a Buehler Phoenix Beta machine to a roughness of Ra = 0.2 ÷ 0.8 μm.

The total testing time of each sample was 165 min, which was divided into 12 periods (5 min, 10 min and 10 periods of 15 min). At the end of each test period, the samples were washed under a jet of water, with alcohol, acetone, and dried under a jet of warm air. This was followed by their weighing with an analytical balance whose precision was 5 significant decimals (up to 0.00001 g), type Zatklady Mechaniki Precyzyjnej WP 1, in order to determine material losses through cavitation erosion.

After each test period, the surface of the samples was photographed using the Canon PowerShot Sx200 IS, 12× Optical Zoom, whose resolution additionally allowed highlighting the extent of damage over the entire surface. At the end of the 165 min of attack, the roughness of degraded surfaces was investigated using the Mitutoyo SJ210 (Japan) device. The topography of the surfaces was examined with a TESCAN VEGA 3 LMU Bruker EDX Quantax scanning electron microscope.

Based on the mass losses Δm_1_, Δm_2_, Δm_3_, from each period “i”, for the set of three samples related to each structural state, the average mass loss was determined, according to the following relationship:


(1) $${\Delta m}_{i}=\sum_{j=1}^{3}\frac{\Delta m_{j}}{3},\;\;\text{where j = 1, 2, 3- is the sample number}$$

The mean cumulative mass loss over a certain cavitation attack time, until completion (165 min), was determined with the relationship:


(2) $$M_{i}=\sum_{i=1}^{12}\Delta m_{i}$$

Based on these losses, following the procedure described in [20,21], the values of the cumulative mean depths MDE_i_ and of the erosion penetration rates MDER_i_ were determined with the relations:

(3) $${\mathrm{MDE}}_{i}=\frac{4\cdot M_{i}}{\rho \cdot \pi \cdot d_{p}^{2}}\;[\mu m]$$

MDER_i_ = ΔMDE_i_/Δt_i_ [µm/min.] (4)

where

i is the testing time;

Δm_i_ is the mass of material, lost through erosion, in period “i”, in grams;

ρ material density, in grams/mm^3^;

Δt_i_ cavitation time corresponding to period “i” (5 min, 10 min, or 15 min);

d_p_ diameter of the sample surface, exposed to cavitation (d_p_= 15.8 mm);

ΔMDE_i_ value of the mean penetration depth of erosion, achieved by cavitation during the period Δt_i_.

## 4. Results and Discussions

### 4.1. Cavitation Curves

**Figure 8:**
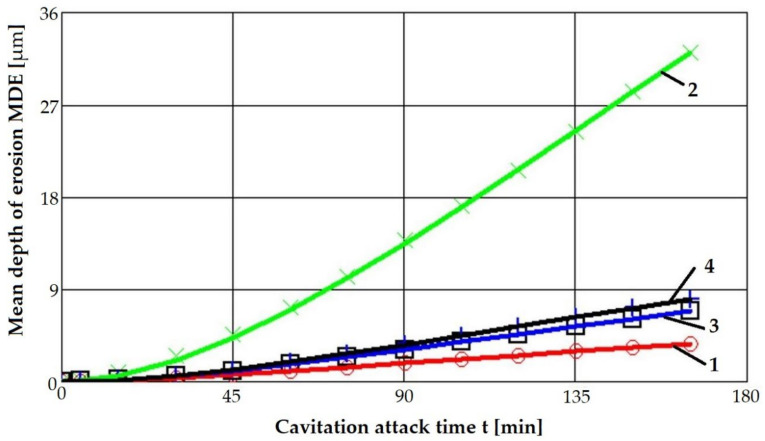
The variation of the mean penetration depth of erosion with the cavitation attack time: 1 nitriding; 2 solution treatment; 3 TIG remelting with Is = 80 A; 4 solution treatment followed by artificial aging.

**Figure 9:**
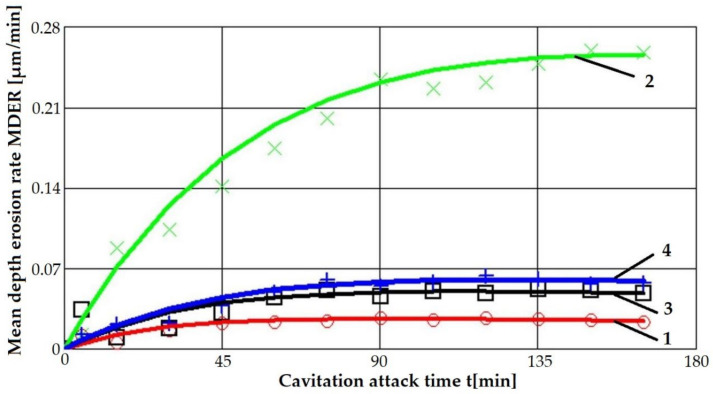
The variation in the mean erosion penetration rate with the cavitation attack time: 1 nitriding; 2 solution treatment; 3 TIG remelting with Is = 80 A; 4 solution treatment followed by artificial aging.

Figure 8 and Figure 9 show the variation curves of these two parameters depending on the cavitation attack duration of the four technological options for modifying the microstructure of the considered alloy.

The main observations derived from the analysis of these curves are as follows:

- The highest values of the mean penetration depth of erosion, MDE, are characteristic of the solution treatment;
- The lowest values of MDE were obtained after applying the thermochemical nitriding treatment;
- The local modification by TIG remelting (Is = 80 A) of the material surface led to lower cavitation losses compared to the heat treatment composed of a solution followed by artificial aging (MDE = 6865 µm).

According to the international norm ASTM G32—2016, the inverse of the other cavitation parameter towards which it tends to stabilize, MDERs, defines the resistance to cavitation, R_cav_.

Table 2 shows its values, presented comparatively for the processing conditions applied to the material.

The data from this table highlights an increase of approximately 4…10 times the cavitation resistance of the strengthened surfaces using the applied techniques.

### 4.2. Surface Hardness Measurements

**Figure 10:**
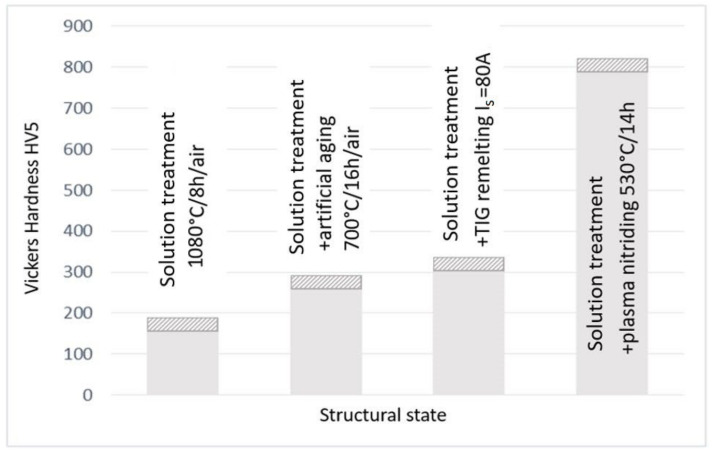
Histogram of surface hardness values.

Since hardness is the mechanical property most sensitive to structural changes in a metallic material, the samples processed by the four technological variants were subjected to such measurements. On the front surface of these samples, eight Vickers hardness measurements were made with a load of 50 N. Based on the obtained results, the histogram shown in Figure 10 was drawn. These data demonstrate that there is a full correspondence between the hardness and the material’s resistance cavitation erosion degradation. The lowest hardness values are specific to solution heat treatment (approx. 181 HV5), and they correspond to the highest erosion rate (0.255 µm/min). Instead, the thermochemical plasma nitriding treatment ensures a high hardness (approx. 800 HV5), which favors a pronounced decrease in the erosion rate (0.024 µm/min). The high values of the hardness are attributed to the microstructure generated in the nitrided layer, consisting of carbonitride particles embedded in a Ni-based γ solid solution matrix.

### 4.3. Roughness of the Cavitation Tested Surfaces

**Figure 11:**
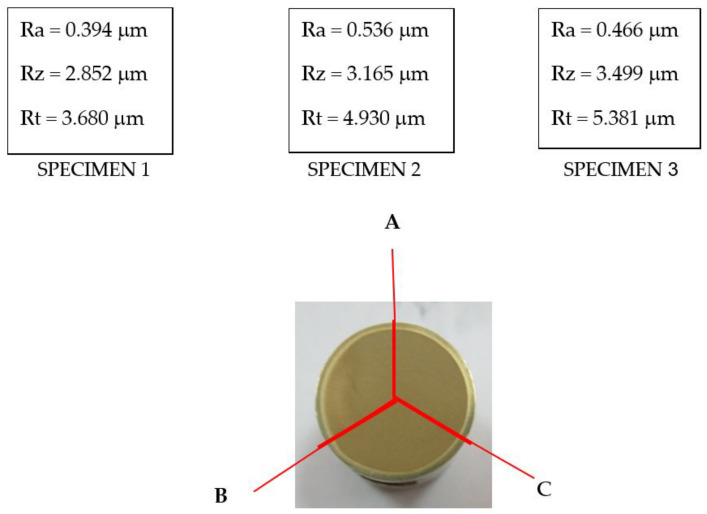
Roughness measurement mode with the Mitutoyo SJ210 device at minute 165.

The samples surfaces subjected to the four technological variants of processing and tested for cavitation erosion for 165 min were roughness measured using the Mitutoyo SJ210 device. Figure 11 exemplifies the three-way measurement of the roughness Ra, Rz, and Rt for the state of nitriding thermochemical treatment. Table 3 summarizes their mean roughness results together with the values of mean erosion penetration depth for the 4 surface ennobling techniques of the Nimonic 80A alloy. The comparative analysis of the results obtained (Table 3) highlights the differences between the solution-treated state and the structural state obtained after applying the other three thermal processing processes. They prove that by applying the thermochemical plasma nitriding, the greatest increase in cavitation erosion resistance is obtained.

### 4.4. Structural Analyses

**Figure 12:**
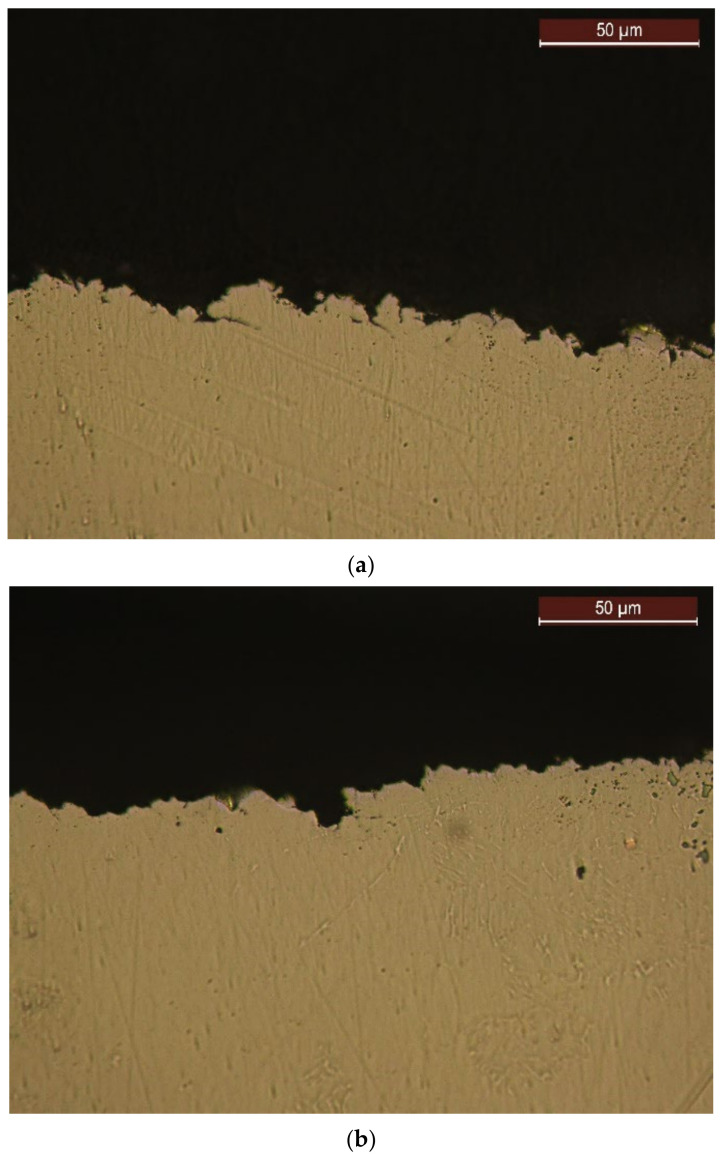

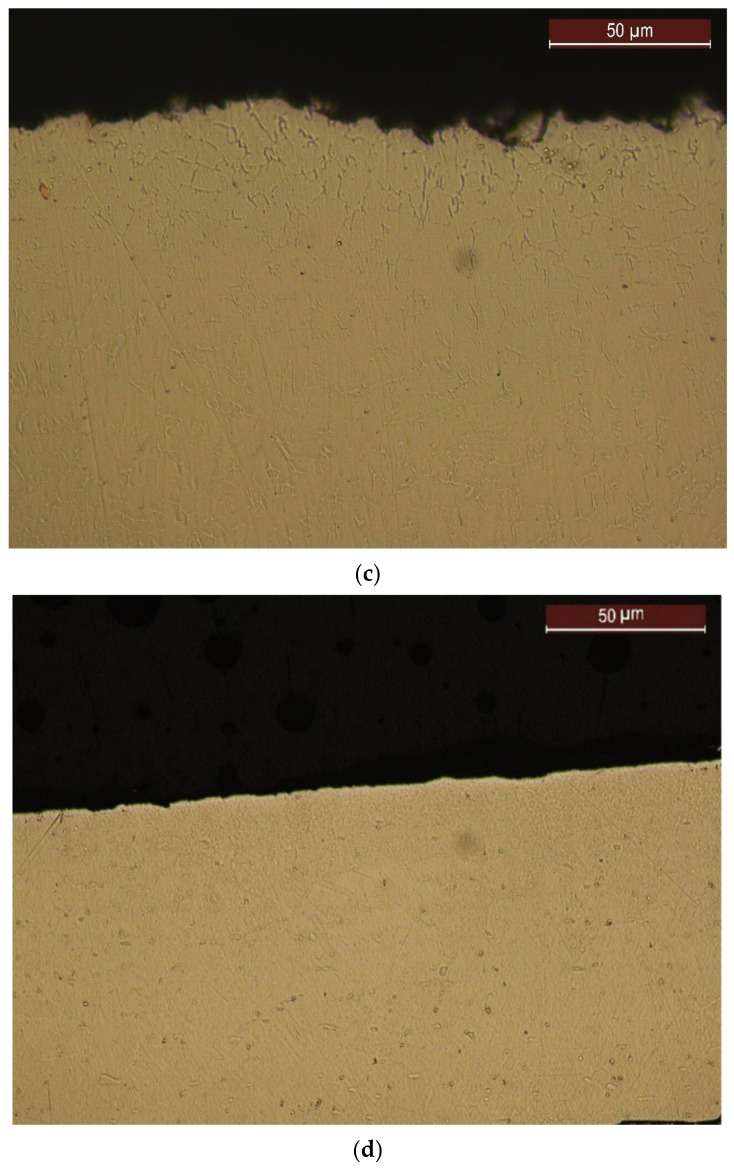
The images of some cross sections through the cavitated layers after 165 min: (**a**) solution treatment; (**b**) solution treatment followed by artificial aging; (**c**) solution treatment followed by TIG remelting; (**d**) solution treatment followed by nitriding.

Figure 12 shows images of some cross-sections through the surface layer of the samples processed by the four technological variants and tested by cavitation for 165 min. In the unetched state, the differences are revealed regarding the intensity of the surface cavitation erosion phenomenon, which are specific to each processing process. The greatest irregularities (Figure 12a) are identified on the material surface subjected to the solution treatment, which leads to the lowest hardness values, respectively, to the highest values of the cavitational parameters, MDE and MDER. On the other hand, the lowest surface degradation (Figure 12d) occurs on surfaces strengthened by plasma nitriding.

**Figure 13:**
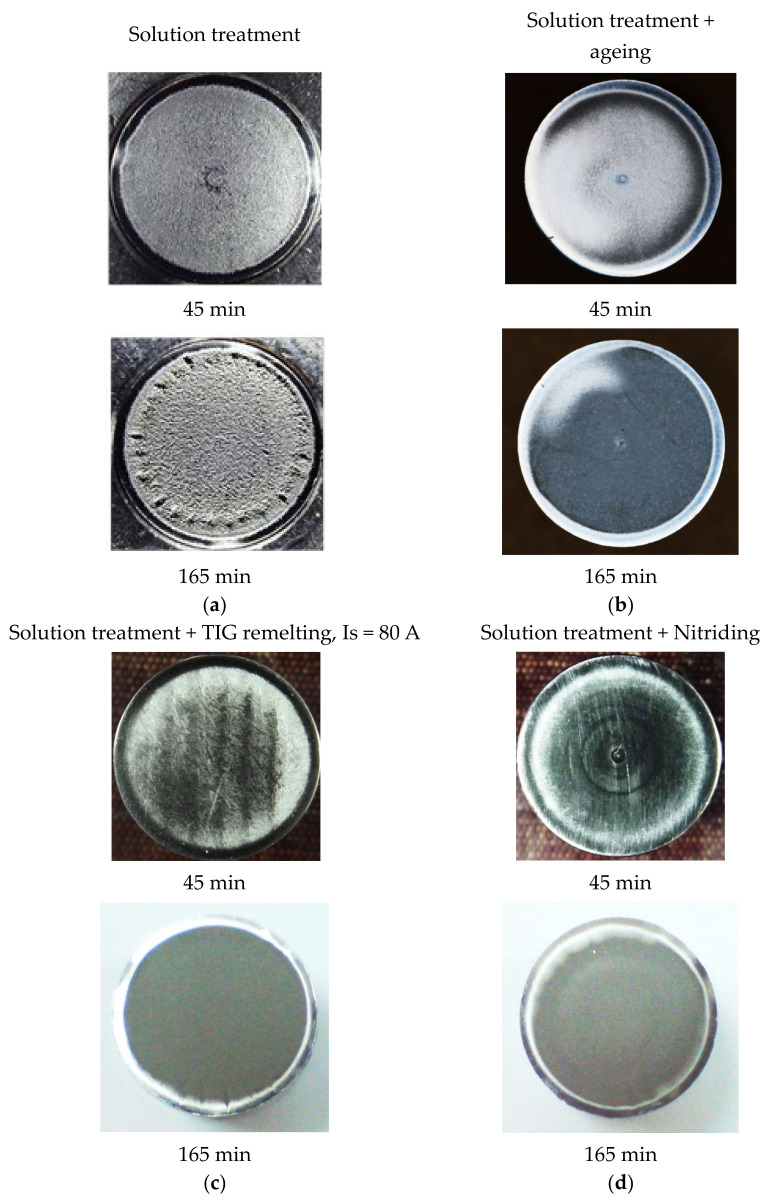
The macrographic images of the degraded surfaces after 45 min and 165 min of cavitation: (**a**) solution treatment; (**b**) solution treatment followed by artificial aging; (**c**) solution treatment followed by TIG remelting with Is = 80 A; (**d**) solution treatment followed by nitriding.

The same observations can be derived from the macroscopic images of the degraded surfaces by ultrasonic cavitation for 45 min and 165 min (Figure 13). It should be noted that after the application of the solution treatment, the surface degradation is visible even after the first 45 min of cavitation attack and is pregnant at the end of the 165 min test period (Figure 13a). Instead, the other three processes of the material structure modification through which the hardness increases more and more are manifested by a less intense surface degradation throughout the cavitation erosion attack (Figure 13b–d). The morphology of the structural transformations involved in the layer-substrate system before and after the cavitation erosion tests have been analyzed in scientific works [17,18].

**Figure 14:**
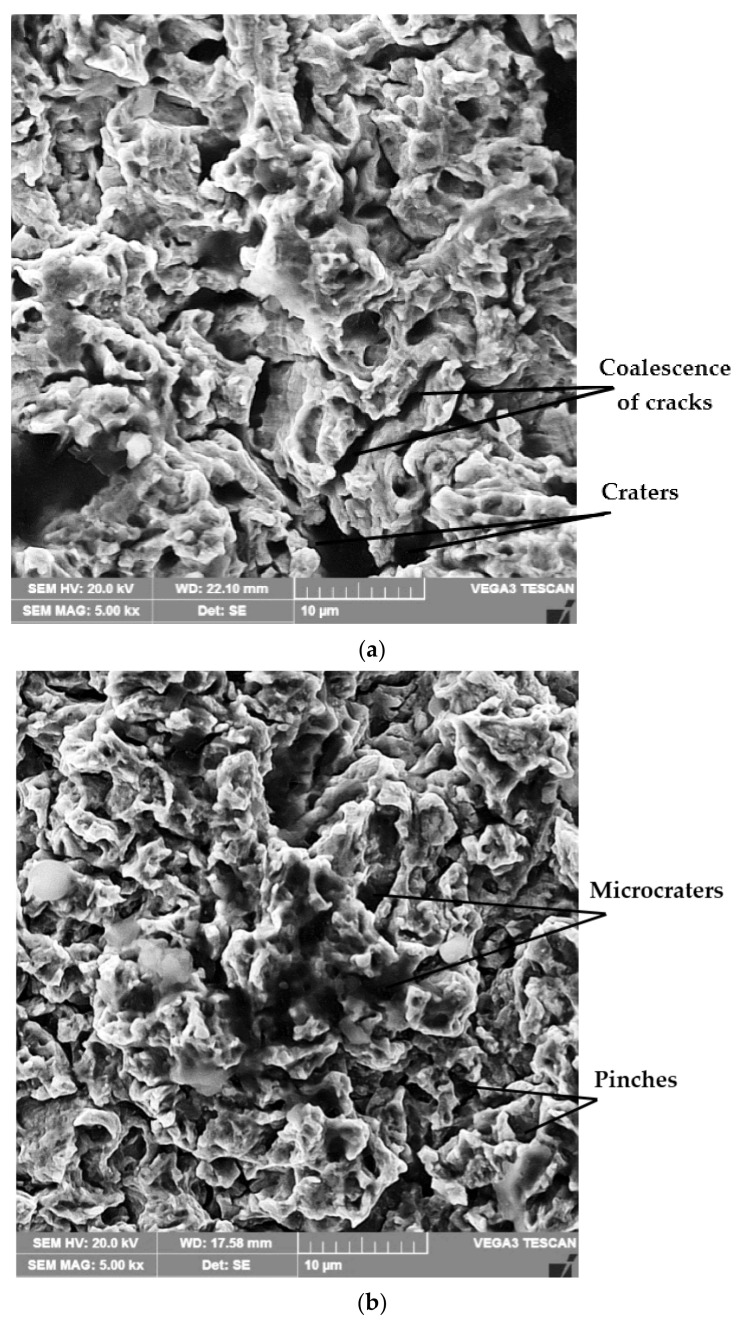

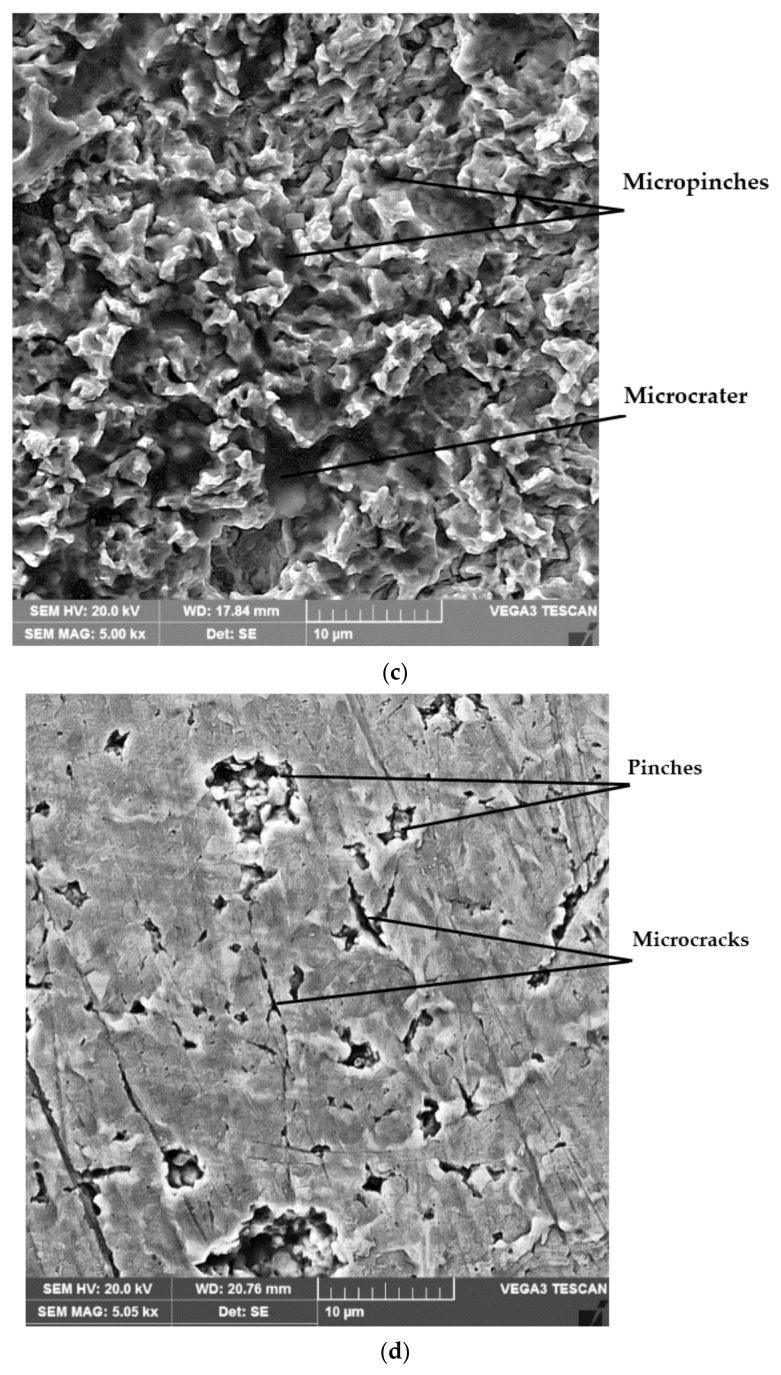
The microfractographic images of the degraded surfaces at 165 min of cavitation: (**a**) solution treatment; (**b**) solution treatment followed by artificial aging; (**c**) solution treatment followed by TIG remelting with Is = 80 A; (**d**) solution treatment followed by nitriding.

Scanning electron microscope examination of the topography of the surfaces treated differently and tested for cavitation (Figure 14a–d) proves that the material removal mechanisms, during cavitation erosion, are controlled by a plastic deformation process, accompanied by the formation of sliding steps, characteristic of the solid solution matrix with a nickel base (γ), which has an fcc crystalline lattice. From their analysis, it can be seen that in the samples subjected to the solution treatment, the intensity of the surface degradation phenomenon is at a maximum, with the boundaries between the grains being preferentially attacked by microcracking followed by the expulsion of the crystalline grains. The topographic image of the samples surface from the reference material (Figure 14a) highlights the formation of craters whose depths are greater than 20 µm, determined by the preferential cavities of the boundaries between the grains of the solid solution γ, which are more fragile. It is emphasized that the boundaries between the grains are less able to absorb the deformation energy due to the stresses induced in the material by the cavitation impact waves, and as a result, they erode faster than the solid solution microstructure γ.

In the case of the samples treated by the solution followed by artificial aging, the nucleation of fatigue cracks occurred on the boundaries between the solid solution grains γ and the precipitated secondary phases, i.e., in the areas with stress concentrators. Interfaces cannot undergo mechanical strengthening and consequently become brittle, and they fragment due to cavitation attack. At the same time, the localized strength of the structural matrix increased with the degree of cold deformation during cavitation erosion. This explains why the boundaries between the grains were attacked more compared to the γ solid solution grains in the studied alloy. The pinches that initially formed underwent the phenomenon of coalescence over time and gave rise to microcracks. As the test time increased, the attacked area of the samples became larger, with more micro-pinching, pinching, and subsequent deformation of the solid solution matrix. The removal of the material through the coalescence of these pinches led to the formation of microcraters, as can be seen in Figure 14b.

A similar situation from a qualitative point of view also occurs in the samples whose surface was locally remelted using the TIG technique, in the sense that the initiation of cracks occurred in the area of the interfaces between the solid solution grains γ and the particles of chemical combinations (Figure 14c), with poor mechanical resistance characteristics. Finishing the grain and limiting the precipitation of secondary phases after TIG surface remelting increased the energy absorption capacity of the cavitation impact wave, which delayed the nucleation of cracks.

By plasma-nitriding samples, the crack initiations were determined by the nitride particles and the separation limits between them, as well as the separation limits between the grains of solid solution alloyed with nitrogen. The high-hardness microstructure of the marginal layer causes small and uniform wear with fine nicks without the appearance of deep craters. The pinches present in the eroded surface, especially on the boundaries of the grains of solid solution γ are the places of the former nitride particles of the alloying elements. Increasing the nitrogen content in the surface layer increases the elastic energy returned to the cavitation medium and decreases the amount of plastic energy absorbed by the alloy in the impact points of the cavitation. The cavitation depths do not exceed 10 µm, and the values of the roughness parameters decrease by approx. 10 times. The microcraters present on the eroded surface are the places of the former nitride particles of the alloying elements.

## 5. Conclusions

Compared to the solution treatment, the application of artificial aging caused a 4.3 times increase in the cavitation erosion resistance.

Ennobling the surface by local TIG remelting (Is = 80 A) or by thermochemical plasma nitriding treatment ensured an increase in cavitation erosion resistance by 5.2 and 10.6 times, respectively.

The low hardness values (approx. 181 HV5), which are specific to the solution treatment, are responsible for the highest erosion rates (0.255 µm/min.) and the high hardness values (approx. 800 HV5), characteristic of the thermochemical plasma nitriding treatment, which favors a pronounced decrease in the erosion rate (0.024 µm/min.).

The microstructure resulting from nitriding, which has a high hardness, suffers a slower and uniform degradation with extremely fine pinches.

Although the nitriding treatment involves additional costs, it is fully justified due to the extension of the life of the components that work in cavitation conditions and the reduction in maintenance times and costs during the operation period.

The topography of the cavitation erosion surfaces highlights the presence of caverns arranged towards the periphery of the solution-treated samples, which have a lower hardness and a slower and uniform degradation with the finishing of the crystalline grains and with the increase in the strengthening degree through the applied techniques (TIG and plasma nitriding).

## Figures and Tables

**Table 1:** Chemical composition of Nimonic 80A alloy, wt.%.

Chromium	20.5
Titanium	1.98
Aluminum	1.54
Iron	2.14
Cobalt	1.61
Manganese	0.63
Silicon	0.45
Copper	0.12
Carbon	0.08
Sulfur	0.014
Zirconium	0.11
Nickel	Balance

**Table 2:** The effect of treatment processes on the cavitation erosion parameters.

Structural State	Cavitation Erosion Resistance Parameter		Variation Compared with Solution Treated State
	MDER_s_ (µm/min)	R_cav_. (min/µm)	
Solution treatment	0.255	3.92	-
Solution treatment followed by artificial aging	0.059	16.94	Increase of 4.3 times
Solution treatment followed by local TIG remelting, Is = 80 A	0.049	20.4	Increase of 5.2 times
Solution treatment + nitiding	0.024	41.67	Increase of 10.6 times

**Table 3:** Mean values for MDE and roughness parameters.

Structural State	MDE (165 min.)	Ra, µm	Rz, µm	Rt, µm
Solution treatment	32.11	5.539	31.598	48.026
Solution treatment + artificial aging	8.068	1.975	12.504	17.306
TIG remelting, Is = 80 A	6.86	1.659	10.507	14.543
Nitriding	3.71	0.465	3.172	4.663

## Data Availability

Not applicable.
